# Supplementary material for: Multi‐dataset identification of innovative feature genes and molecular mechanisms in keratoconus
Source: J Cell Mol Med. 2024 Sep 19;28(18):e70079. doi: 10.1111/jcmm.70079 (PMC11412914; doi:10.1111/jcmm.70079)
Supplement: Supplementary file 2 — Material S2. [file JCMM-28-e70079-s002.zip › 7.GSEA analysis/6.SNORA74B/KEGG_TASTE_TRANSDUCTION.html]

Details for gene set KEGG\_TASTE\_TRANSDUCTION[GSEA]

|  || Dataset | SNORA74B.group.cls#h\_versus\_l |
| Phenotype | group.cls#h\_versus\_l |
| Upregulated in class | h |
| GeneSet | KEGG\_TASTE\_TRANSDUCTION |
| Enrichment Score (ES) | 0.31225824 |
| Normalized Enrichment Score (NES) | 0.9003848 |
| Nominal p-value | 0.6204239 |
| FDR q-value | 1.0 |
| FWER p-Value | 1.0 |
Table: GSEA Results Summary

  

Fig 1: Enrichment plot: KEGG\_TASTE\_TRANSDUCTION      
 Profile of the Running ES Score & Positions of GeneSet Members on the Rank Ordered List

  

| SYMBOL | TITLE | RANK IN GENE LIST | RANK METRIC SCORE | RUNNING ES | CORE ENRICHMENT || 1 | TAS2R10 | na | 513 | 0.597 | 0.0369 | Yes |
| 2 | TAS2R14 | na | 955 | 0.495 | 0.0671 | Yes |
| 3 | TAS2R38 | na | 1114 | 0.471 | 0.1034 | Yes |
| 4 | TAS2R46 | na | 1580 | 0.409 | 0.1254 | Yes |
| 5 | TAS2R19 | na | 1964 | 0.373 | 0.1468 | Yes |
| 6 | TAS2R42 | na | 2181 | 0.356 | 0.1714 | Yes |
| 7 | TAS2R45 | na | 2544 | 0.330 | 0.1895 | Yes |
| 8 | TAS2R20 | na | 2625 | 0.324 | 0.2153 | Yes |
| 9 | GRM4 | na | 3462 | 0.279 | 0.2154 | Yes |
| 10 | TAS2R13 | na | 3924 | 0.262 | 0.2249 | Yes |
| 11 | TAS2R31 | na | 3939 | 0.262 | 0.2472 | Yes |
| 12 | TAS2R43 | na | 4265 | 0.251 | 0.2595 | Yes |
| 13 | TAS2R1 | na | 4465 | 0.244 | 0.2749 | Yes |
| 14 | TAS1R3 | na | 4582 | 0.239 | 0.2923 | Yes |
| 15 | TAS2R50 | na | 4608 | 0.239 | 0.3123 | Yes |
| 16 | TAS2R4 | na | 5502 | 0.208 | 0.3046 | No |
| 17 | ADCY4 | na | 7191 | 0.157 | 0.2695 | No |
| 18 | TAS2R8 | na | 8064 | 0.134 | 0.2560 | No |
| 19 | SCNN1A | na | 9131 | 0.110 | 0.2348 | No |
| 20 | TRPM5 | na | 9369 | 0.106 | 0.2371 | No |
| 21 | PLCB2 | na | 9573 | 0.101 | 0.2400 | No |
| 22 | TAS2R60 | na | 10787 | 0.078 | 0.2118 | No |
| 23 | TAS2R40 | na | 11276 | 0.070 | 0.2038 | No |
| 24 | ADCY6 | na | 12561 | 0.052 | 0.1713 | No |
| 25 | TAS1R1 | na | 16035 | 0.014 | 0.0724 | No |
| 26 | ITPR3 | na | 16729 | 0.007 | 0.0531 | No |
| 27 | ADCY8 | na | 18678 | -0.014 | -0.0019 | No |
| 28 | TAS2R9 | na | 19343 | -0.021 | -0.0192 | No |
| 29 | GNAS | na | 20567 | -0.035 | -0.0515 | No |
| 30 | CACNA1B | na | 21005 | -0.040 | -0.0606 | No |
| 31 | PRKX | na | 21076 | -0.041 | -0.0591 | No |
| 32 | GNG13 | na | 21847 | -0.050 | -0.0770 | No |
| 33 | GNB1 | na | 22177 | -0.054 | -0.0818 | No |
| 34 | TAS1R2 | na | 22425 | -0.057 | -0.0840 | No |
| 35 | PRKACA | na | 22659 | -0.060 | -0.0855 | No |
| 36 | GNG3 | na | 23659 | -0.075 | -0.1077 | No |
| 37 | TAS2R3 | na | 23830 | -0.078 | -0.1059 | No |
| 38 | TAS2R5 | na | 25401 | -0.105 | -0.1420 | No |
| 39 | CACNA1A | na | 25928 | -0.115 | -0.1472 | No |
| 40 | GNAT3 | na | 26444 | -0.126 | -0.1511 | No |
| 41 | TAS2R41 | na | 28001 | -0.165 | -0.1817 | No |
| 42 | TAS2R39 | na | 28471 | -0.179 | -0.1798 | No |
| 43 | TAS2R16 | na | 28686 | -0.185 | -0.1699 | No |
| 44 | GNB3 | na | 30930 | -0.264 | -0.2117 | No |
| 45 | SCNN1B | na | 31263 | -0.281 | -0.1969 | No |
| 46 | PRKACB | na | 32349 | -0.344 | -0.1984 | No |
| 47 | KCNB1 | na | 32450 | -0.351 | -0.1708 | No |
| 48 | PRKACG | na | 32698 | -0.368 | -0.1461 | No |
| 49 | TAS2R7 | na | 33116 | -0.405 | -0.1230 | No |
| 50 | ASIC2 | na | 33539 | -0.454 | -0.0958 | No |
| 51 | SCNN1G | na | 34533 | -0.718 | -0.0622 | No |
| 52 | PDE1A | na | 34593 | -0.782 | 0.0039 | No |
Table: GSEA details [plain text format]

  

Fig 2: KEGG\_TASTE\_TRANSDUCTION      
 Blue-Pink O' Gram in the Space of the Analyzed GeneSet

  

Fig 3: KEGG\_TASTE\_TRANSDUCTION: Random ES distribution      
 Gene set null distribution of ES for **KEGG\_TASTE\_TRANSDUCTION**
